# Erianin facilitates pyroptosis in endometrial cancer via targeting m6A reader YTHDF1

**DOI:** 10.1186/s13020-025-01313-9

**Published:** 2026-04-07

**Authors:** Wan Shu, Xing Zhou, Rong Zhao, Kejun Dong, Xiaoyu Shen, Guanxiao Chen, Shuangshuang Cheng, Qi Zhang, Ting Zhou, Jiarui Zhang, Tangansu Zhang, Shuyang Yu, Haojia Li, Yuwei Yao, Yan Liu, Jun Zhang, Hongbo Wang

**Affiliations:** Department of Obstetrics and Gynecology, Union Hospital, Tongji Medical College, Huazhong University of Science and Technology, Wuhan, Hubei 430022 PR China

**Keywords:** Erianin, Pyroptosis, Endometrial cancer, YTHDF1, FOXM1

## Abstract

**Background:**

Endometrial cancer (EC) is a gynecological malignancy that originates from the endometrial epithelium and has a poor prognosis when advanced, recurrent, or metastatic. The limited therapeutic efficacy and severe adverse effects of conventional chemotherapy in advanced EC highlight the urgent need to develop more effective therapeutic drugs. Accumulating clinical evidence has revealed that natural compounds possess pharmacological advantages, including low toxicity and multi-target mechanisms. Erianin is a natural, small-molecule compound isolated from Dendrobium chrysotoxum Lindl that has multiple pharmacological effects. However, the effects of erianin on EC have not been confirmed and its anticancer mechanisms remain unclear.

**Methods:**

Erianin was identified as a potent natural compound against EC through compound library screening. CCK-8 assays, colony formation assays, Edu experiments, and Live/Dead cell staining assays were used to analyze the anti-proliferative activity of erianin. Morphological characteristics, transmission electron microscopy, lactate dehydrogenase release assays, and western blot assays were used to evaluate the activation of pyroptosis. A transcriptome sequencing analysis was conducted to identify the potential mechanism of erianin. Biotin–erianin was synthesized and 20-k human proteome microarray was used to identify its direct targets. Molecular docking and cellular thermal shift assays (CETSA) were used to investigate whether erianin would bind to YTH domain family proteins (YTHDF1). To evaluate the in vivo therapeutic potential of erianin, an EC xenograft model was established and mechanistic investigations incorporating hematoxylin and eosin and immunohistochemical (IHC) staining, and western blot assays were conducted.

**Results:**

Erianin inhibited the cell proliferation of EC cells and promoted pyroptosis through the caspase-3/gasdermin E (GSDME) pathway. Mechanistically, a crucial role for FOXM1/RRM2-mediated DNA damage in erianin-induced pyroptosis was established. Protein microarrays indicated that erianin–biotin directly targeted the m6A reader, YTHDF1. Erianin was confirmed to bind to YTHDF1 using molecular docking and CETSA. Molecular studies indicated that erianin inhibited YTHDF1, recognized m6A-modified FOXM1, and promoted FOXM1 mRNA degradation, which led to DNA damage and caspase-3-mediated GSDME cleavage. Erianin also substantially inhibited EC tumor growth in EC models.

**Conclusion:**

Erianin directly targeted YTHDF1 to suppress the FOXM1/RRM2 axis and consequently promoted caspase-3/GSDME-dependent pyroptosis in EC cells. Our findings provide a new strategy for further clinical exploration of EC.

**Graphical Abstract:**

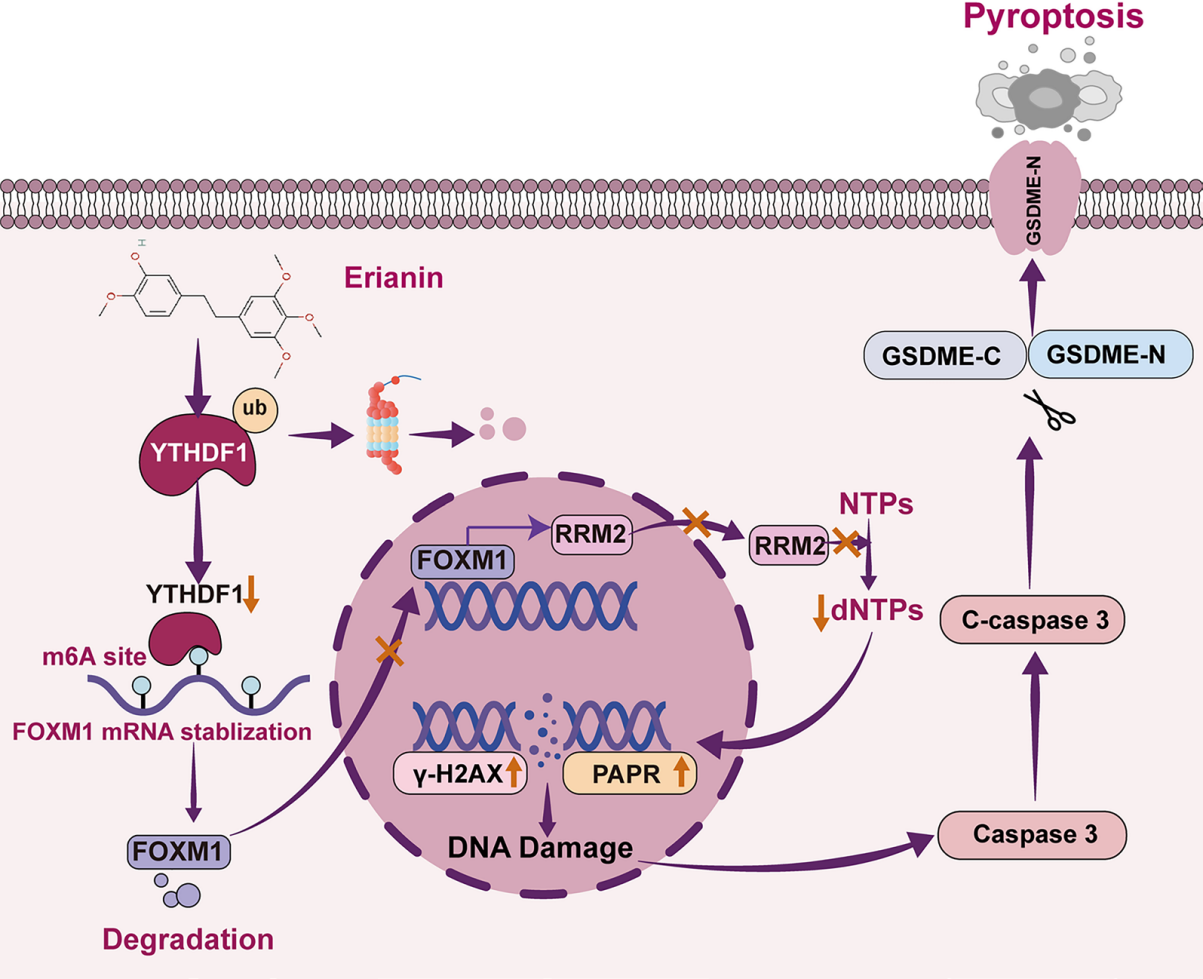

**Supplementary Information:**

The online version contains supplementary material available at 10.1186/s13020-025-01313-9.

## Background

Endometrial cancer (EC) comprises a group of malignant epithelial tumors that arise in the endometrium and consist mostly of adenocarcinomas originating from the endometrial glands [1, 2]. The global morbidity and mortality of EC is increasing [3]. In the United States, 67,880 new cases of EC were diagnosed in 2024, with 13,250 deaths [4]. Compared to 2023, the incidence rate increased by 2% and its mortality growth rate surpassed that of breast, cervical, and ovarian cancers [5]. In China, 84,520 new cases of EC were diagnosed in 2022, with 17,543 deaths [6]. Although the outcomes of early-stage EC can be favorable after surgical intervention, patients at high risk of recurrence (such as those with advanced stage III/IVA EC) typically undergo systemic chemotherapy or combined chemoradiotherapy. However, the 5-y survival rates for high-risk patients after adjuvant chemoradiotherapy or chemotherapy alone are 58–65% [7]. Therefore, novel targeted drugs are essential for improving patient outcomes.

Pyroptosis is an inflammatory mode of programmed cell death characterized by cellular membrane perforation and continuous swelling until membranes rupture and release cellular contents, which triggers a potent inflammatory response [8, 9]. The important roles of pyroptosis in antitumor treatments, including chemotherapy, targeted therapy, and immunotherapy, have been previously verified [10–12]. Some natural, low-toxicity, small-molecule compounds can trigger pyroptosis by activating the caspase-3-dependent cleavage of gasdermin E (GSDME) [13]. The organic compound, triptolide, induces GSDME-dependent pyroptosis by inhibiting mitochondrial hexokinase II in head and neck cancer [14]. Investigating natural compounds that target pyroptosis is a novel anticancer strategy.

The most prevalent RNA modification in eukaryotes is N6-methyladenosine (m6A), which regulates all stages of RNA metabolism to modulate mRNA transcript levels [15, 16]. A “writer” protein complex consisting of methyltransferase-like 3/14 (METTL3/14) and Wilms' tumor 1-associating protein (WTAP) catalyzes m6A, and then the demethylases, fat mass and obesity-associated protein (FTO) and alkylation repair homolog protein 5 (ALKBH5), erase m6A from mRNA [17]. The m6A “readers”, YTH domain family proteins (YTHDF1-3) and insulin-like growth factor 2 mRNA-binding proteins (IGF2BPs), recognize and regulate m6A-modified transcripts [18]. As a key m6A “reader”, YTHDF1 recognizes and binds to m6A modifications on mRNA, primarily enhancing the translation efficiency and regulating the stability of its target mRNAs, thereby playing a critical role in the regulation of gene expression. YTHDF1 has been reported to be overexpressed in various cancers and contributes to tumorigenesis and metastasis through multiple signaling pathways. The progression of ovarian cancer is promoted by YTHDF1, which enhances eukaryotic translation via EIF3C [19]. METTL3 promotes cervical cancer proliferation through YTHDF1-dependent HK2 expression and aerobic glycolysis [20]. YTHDF1 is reported to be highly expressed in EC, serves as an independent prognostic marker, and its expression levels are strongly correlated with the International Federation of Gynecology and Obstetrics (FIGO) stages [21]. Collectively, these findings suggest that YTHDF1 has potential as a therapeutic target in EC. Current research indicates that key enzymes involved in m6A modification influence pyroptosis signaling pathways. WTAP-mediated m6A modification regulates NLRP3/Caspase-1/GSDMD to suppress pyroptosis in colorectal cancer (CRC) [22]. Although YTHDF1 has been proposed to regulate pyroptosis, its specific role and molecular mechanisms in modulating pyroptosis in EC and most tumor types remain unclear.

Low-toxicity, plant-derived, natural compounds have demonstrated unique therapeutic advantages in cancer treatment [23, 24]. Erianin, a natural bibenzyl compound extracted from the precious orchid, Dendrobium officinale, exhibits a range of biological properties, including anti-inflammatory, antioxidant, antiviral, and anti-angiogenic activities [25]. Erianin also possesses potent anticancer effects against multiple tumor types, such as lung, colon, gastric, and breast cancers. Erianin triggers ferroptosis to suppress the progression of lung cancer and CRC [26, 27]. In gastric cancer, erianin inhibits cell growth by modulating the LKB1–SIK2/3–PARD3 signaling axis [28], while in breast cancer, it promotes apoptosis through activation of the PI3K/AKT pathway [29]. Collectively, these findings highlight the potential of erianin as a clinically-promising anticancer agent. Nevertheless, its role in EC and the underlying molecular mechanisms are largely unknown.

Our findings showed that erianin directly binds to YTHDF1 and induces the ubiquitination and degradation of YTHDF1, inhibits YTHDF1 from recognizing m6A-modified FOXM1, promotes the degradation of FOXM1 mRNA, inhibits RRM2-regulated nucleotide metabolism, and leads to DNA damage, which activates caspase3/GSDME-dependent pyroptosis.

## Materials and methods

### Cells and reagents

HEC-1-B, Ishikawa, and KLE cells (Zhongqiao, Shanghai, China) authenticated by short tandem repeat (STR) analysis were grown in a temperature-controlled incubator at 37 °C under a 5% CO2 atmosphere. The cells were cultured in F12 and Minimum Essential Medium (MEM) media (Servicebio, Wuhan, China) supplemented with 10% serum (Gibco, MD, USA) and 1% streptomycin and penicillin (Servicebio, Wuhan, China). A compound library containing 60 natural compounds and erianin was obtained from TargetMol (Boston, MA, USA). The caspase-3 inhibitors, cell-permeable fluoromethyl ketone (Z-DEVD-FMK), cycloheximide (CHX), and chloroquine (CQ), and the proteasome inhibitor, MG132, were obtained from Selleck Chemicals (Houston, TX, USA).

### Cell viability assay

Cell viability was assayed using Cell Counting Kit-8 (CCK-8; C0005; TargetMol, Boston, USA). An amount of 5 × 10^3^ cells/well were seeded into 96-well plates and incubated with or without erianin followed by incubation with 10% CCK-8 reagent for 2 h. Cell viability was determined by measuring the absorbance at 450 nm using a microplate reader.

### Transmission electron microscopy (TEM)

The culture medium was removed, and the cells were fixed in electron microscope fixative (Servicebio, Wuhan, China) for 5 min at room temperature in the dark and then dehydrated. The embedded cell pellets were solidified into blocks at 60 °C for 48 h. Ultrathin sections were stained, and ultrastructural morphology was analyzed using TEM images.

### 5-ethynyl-2'-deoxyuridine (EdU) assay

Cell proliferation was assessed using EdU assay kits (C0075S, Beyotime Biotechnology, Shanghai, China) according to the manufacturer’s protocol. Cells were incubated with 10 μM EdU for 2 h, fixed for 10 min, and then incubated with Click Additive Solution for 2 h in the dark. Finally, nuclei were counterstained with DAPI for 10 min. The fluorescence images were analyzed using fluorescence microscopy.

### Live/dead cell staining assay

The viability of EC cells was analyzed using a live/dead cell kit (Yeasen, China). After incubation with calcein–acetoxymethyl (AM)/propidium iodide (PI) for 0.5 h, fluorescence images were acquired using a fluorescence microscope (Nikon Corporation, Japan). Green fluorescence (calcein‐AM) represents viable cells, and red fluorescence (propidium iodide) represents dead cells.

### Lactate dehydrogenase (LDH) assay

The LDH release rate was assessed using LDH Cytotoxicity Detection Kits (C0075S, Beyotime Biotechnology, Shanghai, China) according to the manufacturer’s protocol. The supernatant from each sample was incubated with the LDH assay reagent at room temperature, protected from light for 25 min, followed by measurement of the absorbance at 490 nm. The LDH release rate was calculated according to the following formula:

$${\text{LDH release rate}}\, = \,\left( {{\text{Absorbance of treated sample}}\, - \,{\text{Absorbance of untreated control}}} \right) \, / \, \left( {{\text{Absorbance of maximum LDH activity}}\, - \,{\text{Absorbance of untreated control}}} \right)\, \times \,{1}00.$$

### Colony formation

The cells (5 × 10^2^) were cultured for 14 d in six-well plates containing medium refreshed every 3 d, fixed in 4% paraformaldehyde, stained with 0.1% crystal violet (Servicebio), photographed, and counted.

### Western blot

Cells or xenograft tumors (20–40 mg) were lysed in RIPA buffer (Beyotime Biotechnology) supplemented with protease inhibitor and phosphatase inhibitor. Protein concentration was quantified using BCA protein assay kits (Beyotime Biotechnology). Equal amounts of protein were denatured in 5 × loading buffer (Servicebio), resolved by SDS‒PAGE, and transferred to polyvinylidene difluoride membranes. Nonspecific antigen binding was blocked with 5% fat-free milk in TBST for 2 h at room temperature, after which the samples were incubated overnight at 4 °C with primary antibodies against target proteins. The following antibodies were obtained from the respective suppliers: 1:1,000-diluted anti-GSDME (Abcam, ab215191), anti-GSDMD (Abcam, ab209845), anti-caspase-3 (ABclonal, A19654), anti-cleaved caspase-3 (CST,9664), anti-phospho-histone H2A.X (Ser139) (Abmart, TA3187), anti-PARP (ABclonal, A27147), anti-FOXM1 (Proteintech, 13147-1-AP), anti-RRM2 (Proteintech, 11661-1-AP), anti-GSDMB(CST, 76439), anti-GSDMC(CST, 61921), and anti-YTHDF1 (Proteintech, 17479-1-AP), 1:10,000 diluted anti-β-actin (Proteintech, 66009-1-Ig), and anti-GAPDH (Proteintech,60004-1-Ig). The membranes were incubated overnight with horseradish peroxidase (HRP) -conjugated secondary antibodies (ABclonal, Wuhan, China) for 2 h at room temperature. The protein bands were visualized via an enhanced chemiluminescence (ECL) substrate (Bio-Rad, CA, USA) and imaged using the ChemiDoc system. Band density was quantified using ImageJ software.

### Quantitative real-time polymerase chain reaction (qRT-PCR)

Total RNA extracted via TRIzol reagent (Yeasen, Shanghai, China) was reverse transcribed using Vazyme RT Premix (Vazyme, Nanjing, China), after which qRT‒PCR was conducted using SYBR® Green PCR Master Mix (ABclonal) on a CFX Connect Real-Time PCR System (Bio-Rad, CA, USA). GAPDH was used as a housekeepicng gene. Relative mRNA expression was calculated using the 2-ΔΔCT method. Table 1 shows the sequences of primers used for PCR.

**Table 1:** The list of primers used in this study

Primer name	Forward primer (5'−3')	Reverse primer (5'−3')
Caspase3	CTCTGGTTTTCGGTGGGTGT	CTTCCATGTATGATCTTTGGTTCC
GSDME	ACGACGATGCAGAAGTGTGT	CGGAGAAGGCAGAACTCGAA
YTHDF1	GGGGACAAGTGGGTCTCAAG	AGGGTGTCGCTGTGAAAGC
FOXM1	GGAGCAGCGACAGGTTAAGG	GTTGATGGCGAATTGTATCATGG
RRM2	CACGGAGCCGAAAACTAAAGC	TCTGCCTTCTTATACATCTGCCA
CENPA	TTCCTCCCATCAACACAGTCG	CACACCACGAGTGAATTTAACAC
PLK1	CACCAGCACGTCGTAGGATTC	CCGTAGGTAGTATCGGGCCTC
GAPDH	AGATCCCTCCAAAATCAAGTGG	GGCAGAGATGATGACCCTTTT

### Small interfering (si) RNA transfection

The siRNA oligos (GenePharma, Jiangsu, China) shown in Table 2 or nonspecific siControl (negative control) oligos were transfected into cells using LipoRNAi™ (C0535; Beyotime Biotechnology, Shanghai, China).

**Table 2:** The sequences of siRNAs used in this study

si RNA	Sense (5'−3')
GSDME siRNA 1	GGUGACCUGAUUGCAGUAUTT
GSDME siRNA 2	GCAGCAAGCAGCUGUUUAUTT
CASP3 siRNA 1	GGAACCAAAGAUCAUACAUTT
CASP3 siRNA 2	GCAGCAAACCUCAGGGAAATT
FOXM1 siRNA1	GCUGGGAUCAAGAUUAUUATT
FOXM1 siRNA2	GCCAACCGCUACUUGACAUTT

### Plasmid transfection

The coding sequence of human FOXM1 was cloned and inserted into the CMV enhancer-MCS-3FLAG-SV40-Puro vector (GeneChem, Shanghai, China). The coding sequence of human YTHDF1 was cloned and inserted into the penter-CMV-YTHDF1-SV40-Puro vector. The YTHDF1 short hairpin RNA (shRNA) expression vector (GeneChem, Shanghai, China) was packaged in the hU6-MCS-Ubiquitin-EGFP-IRES-puro vector. The YTHDF1 shRNA sequences used were 5′-CGCCGTCCATTGGATTTCCTT-3′ (shRNA-1) and 5′-TACCTGCTCTTCAGCGTCAAT-3′ (shRNA-2).

### RNA library construction and sequencing

Total RNA was extracted with TRIzol reagent (Invitrogen, CA, USA). Library preparation was performed using Optimal Dual-mode mRNA Library Prep Kit (BGI-Shenzhen, China). A certain amount of RNA was denatured at suitable temperature to open the secondary structure, and mRNA was enriched using oligo (dT)-attached magnetic beads. RNAs were then fragmented with fragmentation reagents. Then first-strand cDNA was generated using random hexamer-primed reverse transcription, followed by a second-strand cDNA synthesis. The synthesized double strand cDNA was subject to end repairment reaction. After cDNA end repairment, a single ‘A’ nucleotide was added to the 3′ ends of the blunt fragments through the A-tailing reaction. The reaction system for adaptor ligation was configured to ligate adaptors with the cDNAs and the library products were amplified using PCR and subjected to quality control. The single-stranded library products were produced via denaturation. The reaction system for circularization was set up to get the single-stranded, cyclized DNA products. Any uncyclized, single-stranded, linear DNA molecules were digested. The final single strand circularized library was amplified with phi29 and rolling circle amplification (RCA) to make DNA nanoball (DNB), which carried more than 300 copies of the initial single- stranded, circularized library molecule. The DNBs were loaded into the patterned nanoarray and PE 100/150-base reads were generated on the G400/T7/T10 platform (BGI-Shenzhen, China).

### Human proteome microarray

A 200 k HuProt human proteome microarray was used to screen for erianin targets. The microarray was immersed in blocking buffer at room temperature for 1 h and then incubated with either 10 μM biotinylated erianin (Ruixi Biological Technology, Xian, China) or free biotin (control) for 1 h. Free proteins were removed, and the microarray was incubated with fluorescence-labeled streptavidin (Cy5-SA) to detect and quantify the amount and affinity of erianin binding. Raw data were generated using GenePix Pro 6 Microarray Acquisition and Analysis software (https://axon-genepix-pro.software.informer.com/6.0/) and background-corrected. Sample signals were normalized using Z-score standardization before comparisons. Proteins with Z scores > 2 in the erianin group and Z scores < 2 in the control (D-biotin) group were considered positive hits.

### Molecular docking

Protein structures were downloaded from the Protein Data Bank (PDB) database (PDB ID: 7QKN). The three-dimensional (3D) structure file of erianin was obtained from the PubChem database. The molecular docking data were analyzed using the Glide module integrated in Schrödinger software. The protein structure was optimized using the Protein Preparation Wizard module, including steps such as water removal and hydrogen addition. Subsequently, the ligand was preprocessed with the LigPrep module to perform energy minimization and generate 3D conformations. Finally, molecular docking was carried out using the standard precision -docking protocol.

### Cellular thermal shift assays (CETSAs)

Target engagement was determined using CETSA, as previously described [30]. The cells were incubated without erianin (control) or with erianin (100 nM) for 2 h, and 50 μL portions of the cells suspended in phosphate-buffered saline (PBS) containing 1 mM phenylmethylsulfonyl fluoride protease inhibitor were distributed into tubes and heated for 3 min at 4 °C intervals from 36 to 68 °C. After three freeze‒thaw cycles in liquid nitrogen, soluble proteins were separated using centrifugation at 12,000 × g for 30 min. Thermal stability was analyzed using immunoblotting.

### Ubiquitination assay

For the ubiquitination analysis, EC cells were treated with 20 µM MG132 and 100 nM erianin or 20 µM MG132 for 6 h. The cells were lysed on ice using immunoprecipitation (IP) lysis buffer (P0013, Beyotime, China). The lysates were incubated with YTHDF1 antibody overnight at 4 °C, followed by addition of protein A/G magnetic beads (HY-K0202, MedChemExpress) and incubation for 4 h at 4 °C. The beads were washed three times with IP lysis buffer, and the proteins were eluted with 1 × SDS sample buffer by boiling for 10 min. The YTHDF1 ubiquitination was detected using western blotting with an anti-ubiquitin antibody (Proteintech, 10,201–2-AP).

### Immunofluorescence

Cells cultured on coverslips were incubated with erianin for 24 h, fixed with paraformaldehyde, and permeabilized with Triton X-100 (P0096; Beyotime Biotechnology, Shanghai, China). Nonspecific antigen binding was blocked with bovine serum albumin (BSA), and the cells were incubated overnight with 1:200-diluted anti-phospho-histone H2A.X (Ser139) (Abmart, Shanghai, China), followed by secondary antibodies. The cells were stained with DAPI and visualized using fluorescence microscopy.

### Comet assay

The comet assay was conducted with a commercial kit (KGA1302-50, KeyGEN Bio TECH, Nanjing, China) in accordance with the manufacturer’s instructions. Slides were prepared with a three-layer agarose structure: a base layer of 100 μL of 1% normal melting point agarose, a middle layer containing 10^4^ cells suspended in 0.7% low melting point agarose, and a top layer of 100 μL of 0.7% low melting point agarose. After solidification at 4 °C for 30 min, the slides were subjected to lysis in Lysis Buffer at 4 °C for 2 h. Electrophoresis was then performed in alkaline electrophoresis buffer (containing 0.186 g EDTA, 6 g NaOH, and 500 mL H₂O) at 20 V for 25 min. Neutralization was carried out using 0.4 mM Tris–HCl buffer (pH 7.5), followed by staining with 10 μL propidium iodide (PI) in the dark for 10 min. Images were acquired using a fluorescence microscope.

### Xenograft tumor model

Ishikawa cells (1 × 10^6^) were suspended in 100 μL of PBS and then subcutaneously injected into the right scapular region of 5-week-old female BALB/c-nu nude mice. When the tumors reached 50‒100 mm^3^, the mice were randomly assigned to groups that were intraperitoneally injected with 200 μl 25 or 50 mg/kg erianin daily. The tumor volume was calculated every 3 d as [length × width^2^/2]. The mice were euthanized by cervical dislocation 15 d later, and the xenograft tumors were excised. Parts of the tumors were fixed in formalin and stained with hematoxylin and eosin (H&E) for histopathological analysis. The blood samples were collected for biochemical analysis. The Ethics Committee at Huazhong University of Science and Technology Union Hospital approved all the animal procedures in this study (Approval ID: 2022–3060).

### Immunohistochemistry (IHC)

Immunohistochemical (IHC) analysis of xenograft tumor and human samples was performed using YTHDF1 primary antibodies (Proteintech, Wuhan, China). EC and normal endometrial tissues were obtained from Union Hospital, Huazhong University of Science and Technology. All participants provided written informed consent. The study was approved by the Institutional Ethics Committee (No. 2020-S218).

### RNA immunoprecipitation (RIP)

YTHDF1-FOXM1 mRNA interaction was detected using Megna RNA Binding Protein Kits (MilliporeSigma, Burlington, MA, USA). Cells were lysed with RIP lysis buffer and then rotary incubated with 5 µL of a YTHDF1 or IgG antibody (Proteintech,30,000–0-AP) at 4 °C overnight, followed by washing with magnetic beads at 4 °C overnight. RNA was extracted using TRIzol reagent, and FOXM1 expression was detected using RT‒qPCR.

### Messenger RNA stability assay

Ishikawa and HEC-1-B cells were transfected with shYTHDF1 and incubated with 5 µg/mL actinomycin D. Then, RNA was extracted to detect FOXM1 mRNA expression at the indicated time points.

### Statistical analysis

All the data were statistically analyzed using GraphPad Prism software v. 8.0. The means of three independent replicates between paired groups were compared using unpaired Student’s t tests, and those of three or more groups were compared using one-way analysis of variance (ANOVA), followed by Tukey’s multiple comparison tests. The results are presented as the means ± standard deviation (SD), and P < 0.05 was considered statistically significant.

## Results

### Erianin inhibited EC cell proliferation

**Fig. 1:**
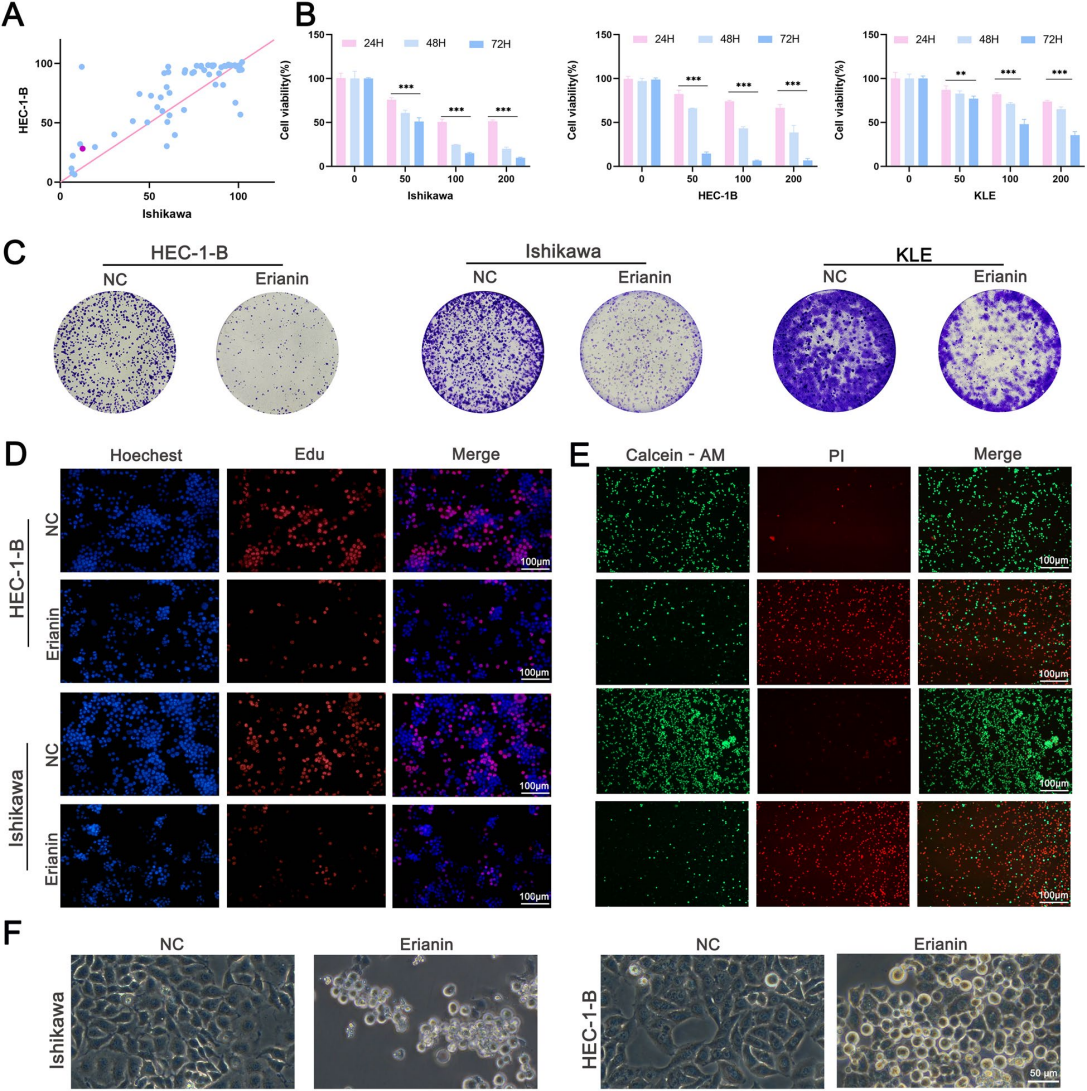
Erianin inhibits EC cell proliferation. **A** 60 natural compound library screening. **B** Viability of Ishikawa, HEC-1-B, and KLE cells incubated with 50, 100, or 200 nM erianin for 24, 48, or 72 h determined using CCK-8 assays. **C** Colony formation of Ishikawa, HEC-1-B, and KLE cells incubated with 100 nM erianin for 24 h. **D** EdU assays of proliferation after a 24 h incubation with 100 nM erianin. Scale bar = 100 μm. **E** Fluorescence microscopy images showing the number of live and dead cells. Green represents live cells and red represents dead cells. Scale bar = 100 μm. **F** Phase-contrast microscopy images showing the morphological characteristics of Ishikawa and HEC-1-B cells. Scale bar = 50 μm. The data are presented as the means ± SD, n = 3; **P < 0.01 and ***P < 0.001 compared with the NC (control group)

Compound library screening is a novel tool for discovering potential anticancer drugs. Small molecule compounds with anticancer efficacy were screened from 60 compounds (Supplementary Table 1). The CCK-8 assays demonstrated that erianin is a promising anti-cancer drug for EC (Fig. 1A). The effects of erianin on the growth of EC cell lines was investigated. HEC-1-B, Ishikawa, and KLE cells were incubated with 50, 100, and 200 nM erianin for 24, 48, and 72 h. The results of the CCK-8 assays indicated that erianin decreased the viability of these cells in a time- and concentration-dependent manner (Fig. 1B). The 100 nM erianin treatment, which substantially inhibited cell proliferation, was selected for the follow-up studies. Erianin substantially reduced the numbers of Ishikawa, HEC-1-B, and KLE colonies compared with those of the controls (Fig. 1C). The EdU findings also revealed that erianin inhibited EC cell proliferation (Fig. 1D). Live/dead cell staining assays indicated that erianin obviously increased the ratio of PI-positive cells (Fig. 1E). Microscopy findings of swollen and rounded cells indicated that erianin inhibited EC cell progression at the nanomolar level (Fig. 1F). Collectively, these data show that erianin inhibits EC cell progression.

### Erianin induced pyroptosis in EC cells via GSDME signaling

**Fig. 2:**
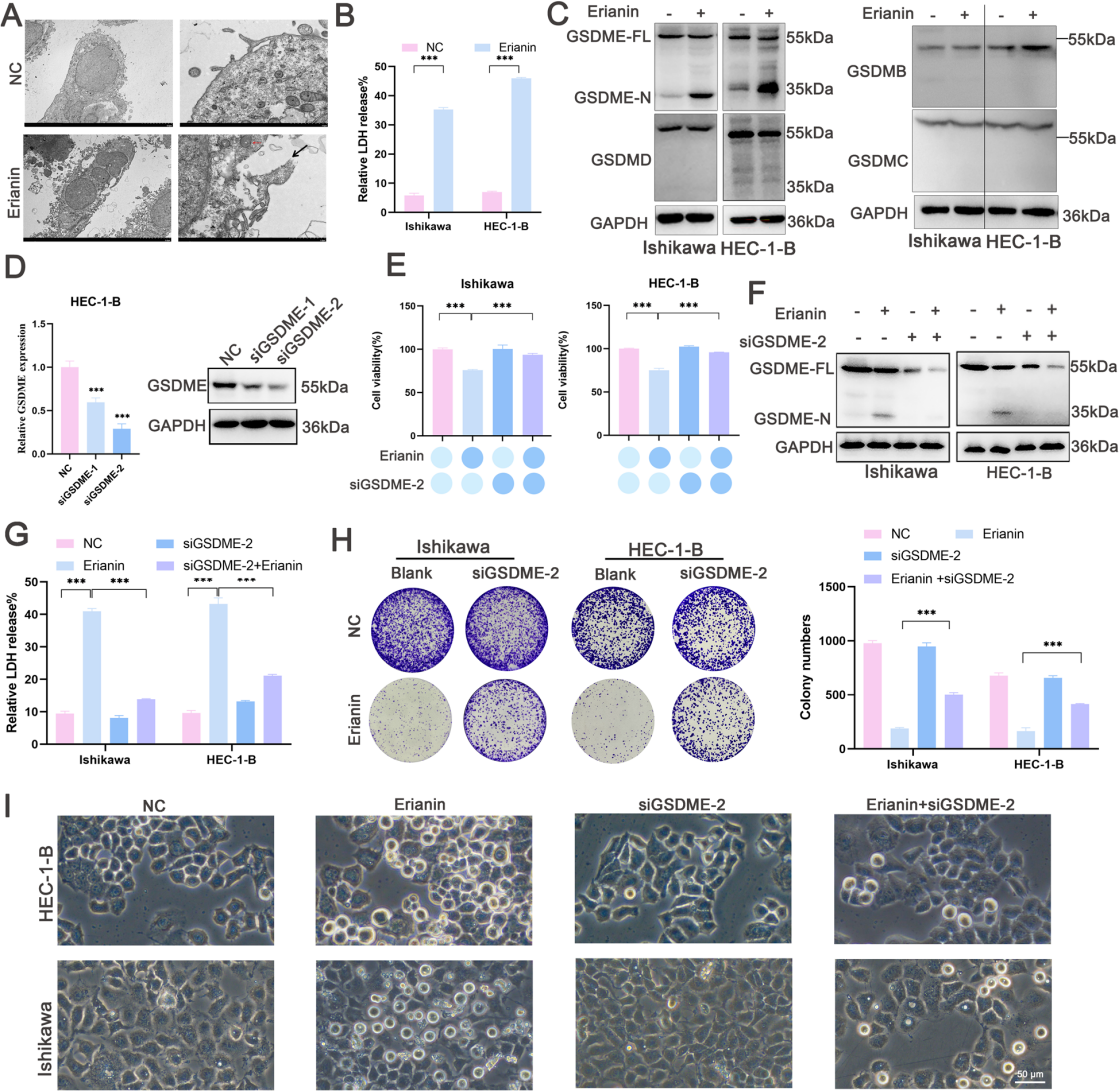
Erianin induces GSDME-dependent EC cell pyroptosis. **A** Transmission electron microscopy (TEM) images of the cell membrane and organelles in HEC-1-B cells incubated with erianin (100 nM). Left and right scale bars, 5 and 1 μm, respectively. Black arrows indicate disruption of the cell membrane, whereas red arrows represent mitochondrial swelling. **B** Release of LDH in HEC-1-B and Ishikawa cells. **C** Western blots of GSDMD, GSDME, GSDMB, and GSDMC in HEC-1-B and Ishikawa cells incubated with erianin (100 nM) for 24 h. **D** qRT‒PCR and western blotting were used to validate the gene expression of GSDME in HEC-1-B cells transfected with si-GSDME. **E**‒**I** GSDME-silenced HEC-1-B and Ishikawa cells were incubated with erianin (100 nM) for 24 h. **E** CCK-8 assays. **F** Western blots of cleaved GSDME. **G** LDH release assays. **H** Colony formation assay. **I** Phase-contrast microscopy of EC cell morphological characteristics. Scale bar = 50 μm. The data are presented as the means ± SD, n = 3; ***P < 0.001 compared with the NC (control group)

Pyroptosis is usually accompanied by cell swelling and membrane rupture. Erianin induced cell swelling and increased the proportion of PI-positive cells. Therefore, we speculated that erianin induced EC cell pyroptosis. The swollen cells had characteristic large bubbles extruding from the plasma membrane according to the TEM images. Substantial membrane disruption was accompanied by mild swelling of mitochondria and irregular shapes of cell nuclei (Fig. 2A). The phenomena closely matched the typical morphological features associated with pyroptosis. Cell rupture is usually accompanied by LDH release, and erianin increased the percentage of LDH release from EC cells (Fig. 2B).

GSDM proteins are key effectors of pyroptosis. Western blotting analysis indicated that erianin induced the cleavage of GSDME rather than GSDMB, GSDMC, and GSDMD, thus yielding a N-terminal fragment that formed pores in the HEC-1-B and Ishikawa cell membranes (Fig. 2C). These findings suggest that erianin induces GSDME-dependent pyroptosis in EC cells. After silencing GSDME using siRNA-mediated knockdown, qRT‒PCR and western blotting analysis indicated that siGSDME-2 reduced GSDME mRNA and protein levels in HEC-1-B cells (Fig. 2D). The CCK-8 results suggested that GSDME knockdown increased the viability of EC cells incubated with erianin (Fig. 2E). Erianin-induced GSDME cleavage (Fig. 2F) and LDH release (Fig. 2G) were inhibited and reduced, respectively, in Ishikawa and HEC-1-B cells. GSDME knockdown resulted in reduced colony formation (Fig. 2H) and EC cell swelling (Fig. 2I). Therefore, silencing GSDME impeded erianin-induced pyroptosis in EC cells, suggesting that GSDME mediated the mechanism through which erianin exerted anticancer effects.

### Erianin induced pyroptosis via caspase-3/GSDME signaling

**Fig. 3:**
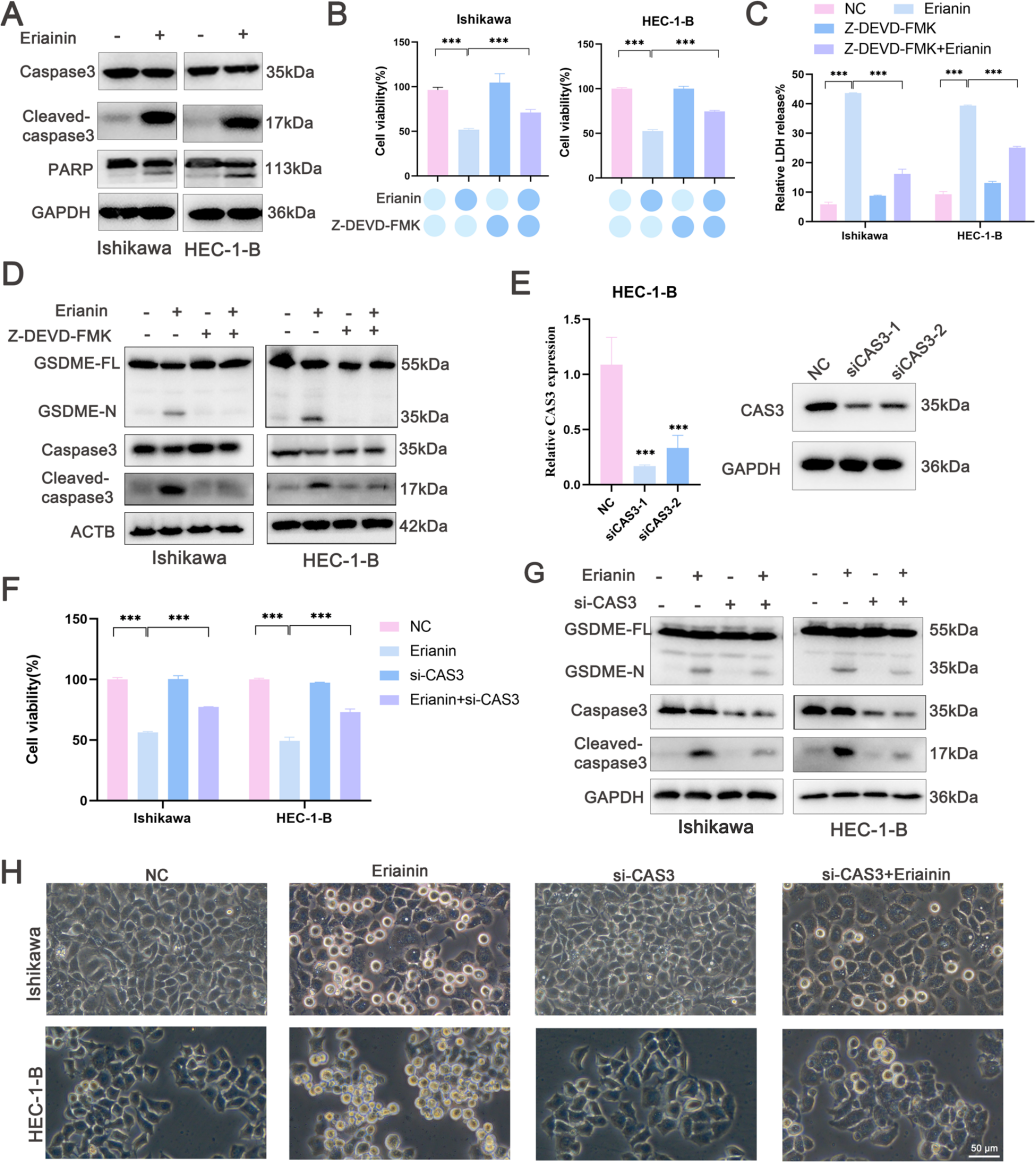
Erianin induces pyroptosis via caspase3/GSDME signaling in EC cells. **A** Western blotting analysis of caspase-3, cleaved caspase-3, and PARP in EC cells incubated with erianin (100 nM) for 24 h. **B**‒**D** HEC-1-B and Ishikawa cells were incubated with or without the caspase-3 inhibitor z-DEVD-FMK (30 μM) for 2 h, followed by erianin (100 nM) for 24 h. **B** CCK-8 assays. **C** LDH release. **D** Western blots. **E** qRT‒PCR and western blotting were used to validate the gene expression of CAS3 in HEC-1-B cells transfected with si-CAS3. **F**–**H** HEC-1-B and Ishikawa cells transfected with si-CAS3 were incubated with erianin (100 nM). **F** CCK-8 assay. **G** Western blot analysis of cleaved caspase-3 and GSDME. **H** Phase-contrast microscopy images showing the morphological characteristics of EC cells. Scale bar = 50 μm. The data are presented as the means ± SD, n = 3; ***P < 0.001 compared with the NC (control group)

Caspase-3 cleaves GSDME during pyroptosis. The levels of cleaved caspase-3 and cleaved PARP were substantially increased in HEC-1-B and Ishikawa cells (Fig. 3A). We therefore investigated whether erianin induced GSDME cleavage through caspase-3 activation. The caspase-3 inhibitor, Z-DEVD-FMK, increased the viability of EC cells incubated with erianin (Fig. 3B) and substantially inhibited erianin-induced LDH release (Fig. 3C), as well as caspase-3/GSDME expression (Fig. 3D). The effects of knocking down caspase-3 were confirmed using qRT‒PCR and western blotting (Fig. 3E). The results of the CCK-8 assays indicated that si-CAS3 increased EC cell survival (Fig. 3F). Western blotting revealed that caspase-3 knockdown inhibited erianin-induced caspase-3 activation and GSDME cleavage (Fig. 3G). Phase contrast microscopy revealed reduced numbers of pyroptotic cells (Fig. 3H). Collectively, these data suggest that erianin-induced GSDME cleavage and subsequent pyroptosis induction required caspase-3 activation.

### Erianin induced DNA damage in EC cells

**Fig. 4:**
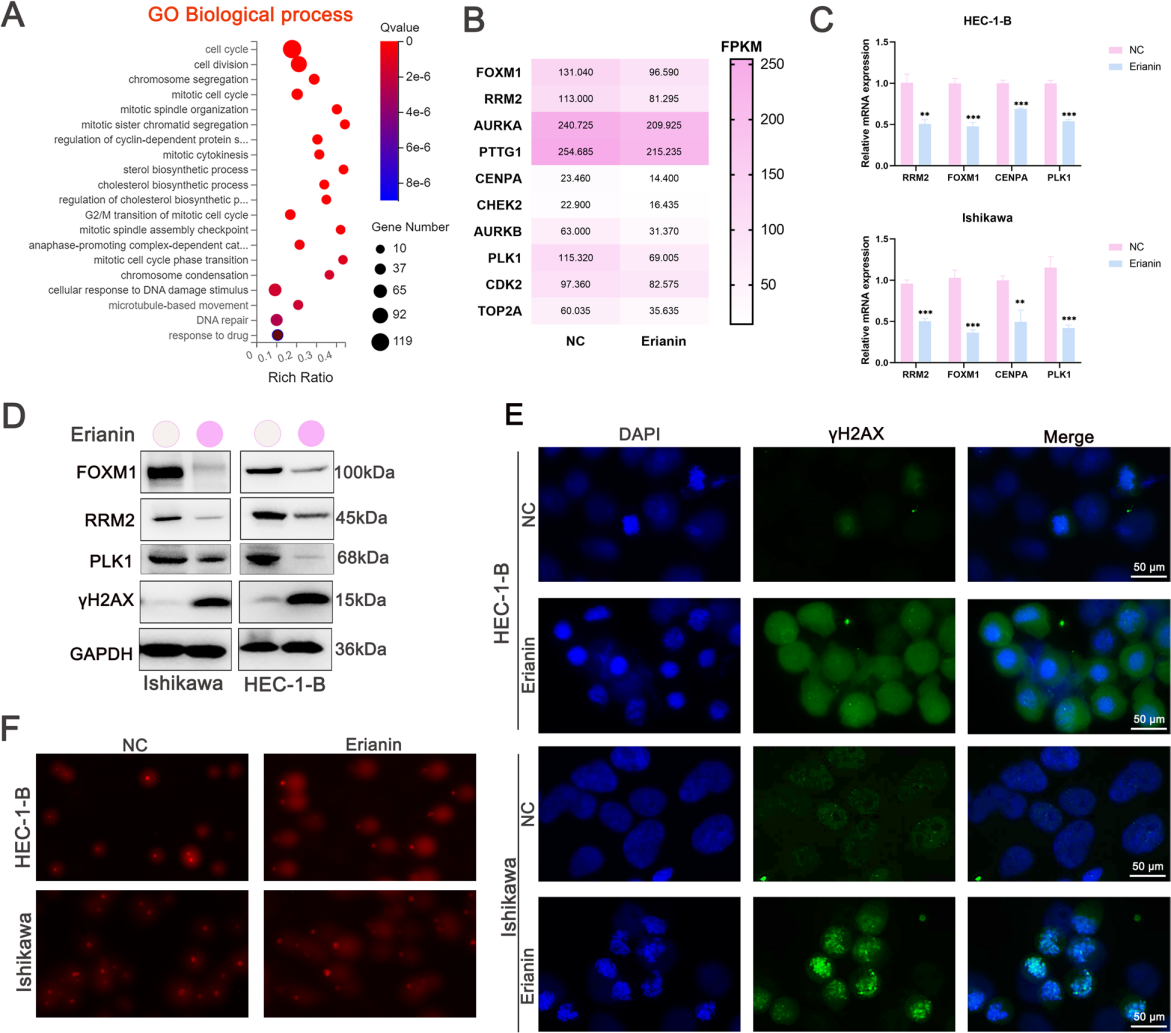
Erianin induces DNA damage in EC cells. **A** Gene Ontology (GO) enrichment analysis of differentially expressed genes (DEGs) in EC cells incubated with erianin (100 nM). **B** Heatmap of DEGs associated with DNA damage. **C** The expression of FOXM1, RRM2, CENPA, and PLK1 in EC cells incubated with erianin (100 nM) for 24 h was detected using qPCR. **D** Western blots of FOXM1, RRM2, PLK1, and γH2AX in Ishikawa and HEC-1-B cells incubated with erianin (100 nM) for 24 h. **E** Immunofluorescence staining of γ-H2AX in HEC-1-B and Ishikawa cells incubated with erianin (100 nM) for 24 h. Scale bar = 50 μm. **F** Comet assay was performed on HEC-1-B and Ishikawa cells treated with 100 nM erianin for 24 h. Scale bar = 50 μm.The data are shown as the means ± SD; n = 3; **P < 0.01 and ***P < 0.001 compared with NC (control group)

The transcriptome was sequenced to further elucidate the mechanism by which erianin induces pyroptosis. Gene Ontology (GO) analysis indicated substantially enriched DNA damage pathways (Fig. 4A). Erianin substantially reduced the expression of the DNA repair factors, Centromere Protein A (CENPA), FOXM1, ribonucleotide reductase M2 (RRM2), polo-like kinase 1 (PLK1), Topoisomerase IIα (TOP2A), Aurora kinase B (AURKB), and Aurora kinase A (AURKA) (Fig. 4B). A close association between pyroptosis and DNA damage has been suggested [31, 32]. Therefore, we further investigated whether erianin promoted DNA damage in EC. The qPCR findings verified that erianin suppressed the expression of FOXM1, RRM2, CENPA, and PLK1 in EC cells (Fig. 4C). The western blotting results further demonstrated that erianin downregulated the expression of FOXM1, RRM2, and PLK1, while increasing the expression of the damage marker H2A.X variant histone (γH2AX) (Fig. 4D). The immunofluorescence staining also showed that erianin increased the number of fluorescent γH2AX foci (Fig. 4E) and elongation of comet tails (Fig. 4F), indicating substantial DNA damage. Therefore, our results suggest that erianin induces DNA damage in EC.

### Erianin induced pyroptosis via the FOXM1/RRM2 axis

**Fig. 5:**
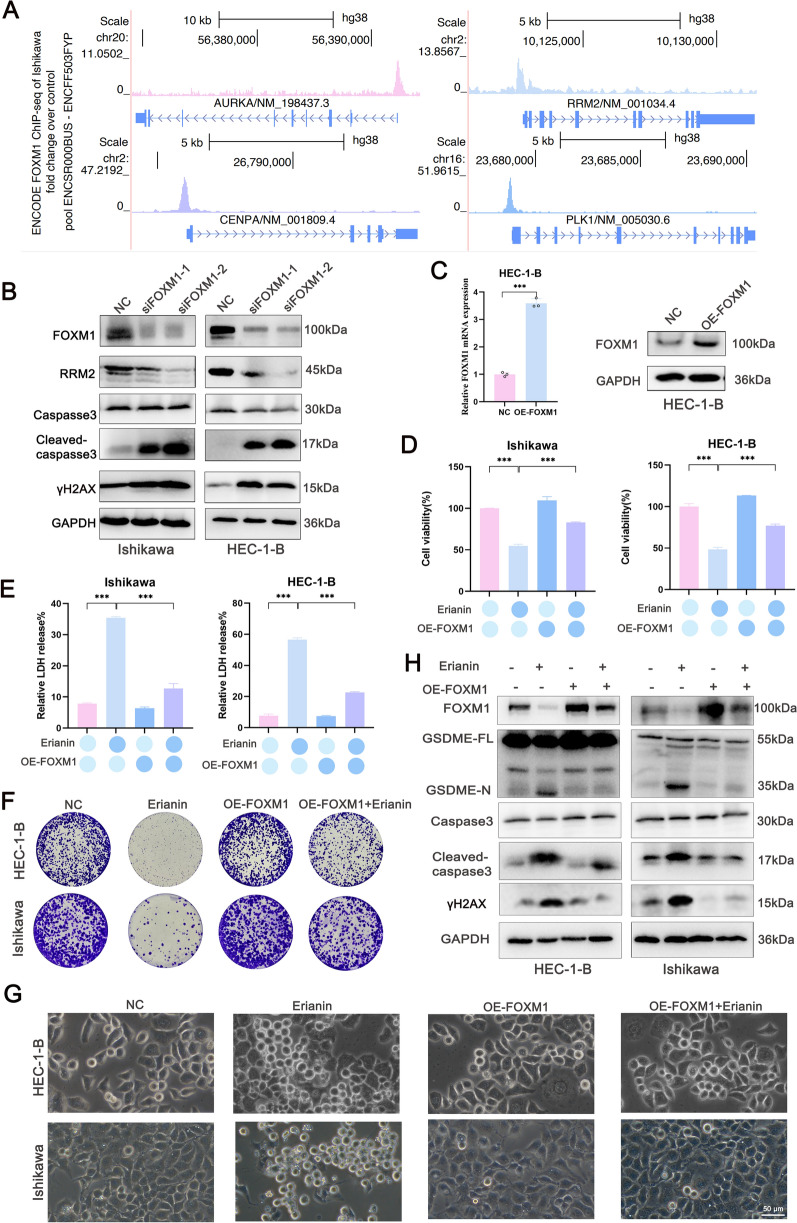
Erianin induces pyroptosis via FOXM1. **A** UCSC Genome Viewer images depicted FOXM1-binding sites in the promoters of CENPA, RRM2, PLK1, and AURKA in Ishikawa cells. **B** Western blots of FOXM1, RRM2, cleaved caspase-3, and γH2AX in Ishikawa and HEC-1-B cells with FOXM1 knockdown. **C** FOXM1 expression in HEC-1-B cells transfected with OE-FOXM1 was validated via qRT‒PCR and western blotting. **D**–**G** FOXM1 was overexpressed in HEC-1-B and Ishikawa cells after incubation with erianin (100 nM) for 24 h. **D** CCK-8 assay. **E** LDH assay. **F** Colony formation. **G** Phase-contrast microscopy of EC cell morphological characteristics. **H** Western blot analysis of cleaved GSDME, caspase3, cleaved caspase-3, and γH2AX. The data are shown as the means ± SD; n = 3; ***P < 0.001 compared with the NC (control group)

Given that analysis of the ENCODE Database showed a shared FOXM1 binding site in the promoters of CENPA, RRM2, PLK1, and AURKA, we hypothesized that FOXM1 functioned as a master transcriptional regulator of these genes (Fig. 5A). FOXM1/RRM2 axis regulates the cell cycle and DNA repair [33]. Western blotting results indicated that FOXM1 knockdown suppressed RRM2 expression while increasing γH2AX and caspase-3 cleavage (Fig. 5B). We further used plasmids overexpressing FOXM1, qRT‒PCR and western blotting assays to further explore whether erianin induces pyroptosis through FOXM1 suppression (Fig. 5C). FOXM1 overexpression increased EC cell viability (Fig. 5D), suppressed LDH release induced by erianin (Fig. 5E), enhanced colony formation (Fig. 5F), and inhibited the proportion of swollen HEC-1-B and Ishikawa cells (Fig. 5G). FOXM1 overexpression also increased caspase-3/GSDME cleavage (Fig. 5H). These results indicate that erianin promotes pyroptosis in EC cells by inhibiting FOXM1.

### Erianin directly targets YTHDF1 and induces YTHDF1 ubiquitination

**Fig. 6:**
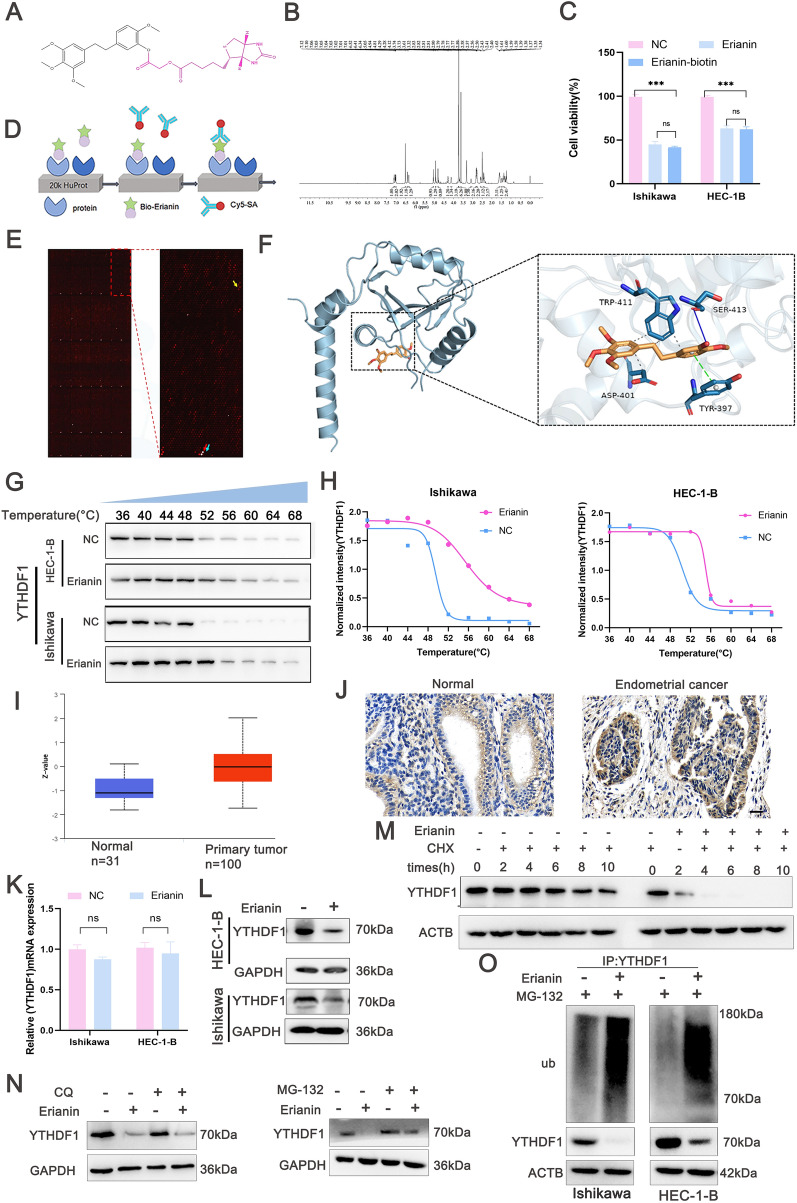
Erianin targets YTHDF1 directly. **A** Synthesis of erianin-biotin. **B** Erianin-biotin 1H NMR spectrum. **C** CCK-8 results for Ishikawa and HEC-1-B cells incubated with erianin-biotin and erianin (50 and 100 nM) for 24 h. **D** Schematic workflow of protein array screening to identify erianin targets. **E** Representative images of the human proteomics chip. Blue, red, and yellow: negative control, positive control, and positive spot, respectively. **F** Molecular docking simulation of the binding between erianin and YTHDF1. **G** Thermal stability of erianin and YTHDF1 in EC cells detected using CESTA. **H** Quantification of YTHDF1 expression. **I** Expression of YTHDF1 in normal endometrium and EC tissues determined from the CPTAC proteomic database. **J** Immunohistochemistry (IHC) staining of YTHDF1 in normal endometrium and EC tissues. Scale bar = 20 μm. (K-N) EC cells were incubated with erianin (100 nM). **K** qRT‒PCR analysis of YTHDF1 expression. **L** Western blots of YTHDF1 expression. **M** Ishikawa cells were treated with erianin (100 nM) and cycloheximide (CHX) (50 mg/mL). **N** Ishikawa cells were treated with MG132 or CQ. **O** Ubiquitination assay of YTHDF1in the lysates from Erianin treated- EC cells in the presence of MG132 (20 μM). The data are shown as the means ± SD; n = 3; ***P < 0.001 compared with the NC (control group). ns, not significant

The targets of erianin that were responsible for DNA damage and pyroptosis were investigated. Conjugation with biotin (Fig. 6A, B) did not impact the anticancer activity of erianin (Fig. 6C). Targets were screened using the 20-k HuProt human proteome microarray (Fig. 6D), which identified the m6A reader, YTHDF1, as a direct target of erianin (Fig. 6E). The crystal structures of YTHDF1 and erianin were compared using molecular docking and erianin was found to bind with Trp-411, Ser-413, Asp-401, and Tyr-397 of YTHDF1 (Fig. 6F). Whether erianin directly targeted YTHDF1 was investigated using CETSA to quantify the binding affinity of small molecules for specific proteins on the basis of changes in protein thermal stability [34]. The CESTA results indicated that incubation with erianin resulted in detectable protein bands of YTHDF1 even at 56 °C (Fig. 6G, H). Thus, erianin improved the thermal stability of YTHDF1, which supported direct binding interactions. YTHDF1 was abundantly expressed in EC cells according to the CPTAC proteomic data (Fig. 6I). To further verify this, we performed IHC staining for YTHDF1 on tumor tissues and normal endometrial tissues collected from patients clinically diagnosed with EC. The IHC results demonstrated that YTHDF1 expression was significantly higher in EC tissues compared to normal tissues (Fig. 6J). The role of YTHDF1 in EC cell proliferation was investigated by using an shRNA targeting YTHDF1. Western blotting and qRT‒PCR confirmed that YTHDF1 was silenced in HEC-1-B cells (Figure S1A). Knockdown of YTHDF1 suppressed cell proliferation (Figure S1B), suggesting that YTHDF1 plays a vital role in EC cell growth. The effect of erianin on YTHDF1 expression was assessed. Erianin reduced YTHDF1 protein expression but did not markedly affect YTHDF1 mRNA in Ishikawa and HEC-1-B cells (Fig. 6K, L). Therefore, we speculated that erianin degraded YTHDF1 mainly at the posttranslational level.

Cycloheximide (CHX) assays indicated a shorter half-life of YTHDF1 in cells treated with both erianin and CHX than in those treated with CHX alone (Fig. 6M). To determine the pathway of YTHDF1 degradation caused by erianin, Ishikawa cells were incubated with erianin and the lysosomal inhibitor, chloroquine (CQ), and the proteasomal inhibitor, MG132. Western blot analysis indicated that MG132, but not CQ, effectively rescued YTHDF1 degradation (Fig. 6N). Additionally, co-immunoprecipitation analysis using anti-YTHDF1 antibody indicated that erianin enhanced the ubiquitination of YTHDF1 in EC cells (Fig. 6O). Collectively, these findings indicate that the ubiquitin‒proteasome pathway is involved in the degradation of YTHDF1 by erianin.

### Erianin-induced EC pyroptosis depends on YTHDF1

**Fig. 7:**
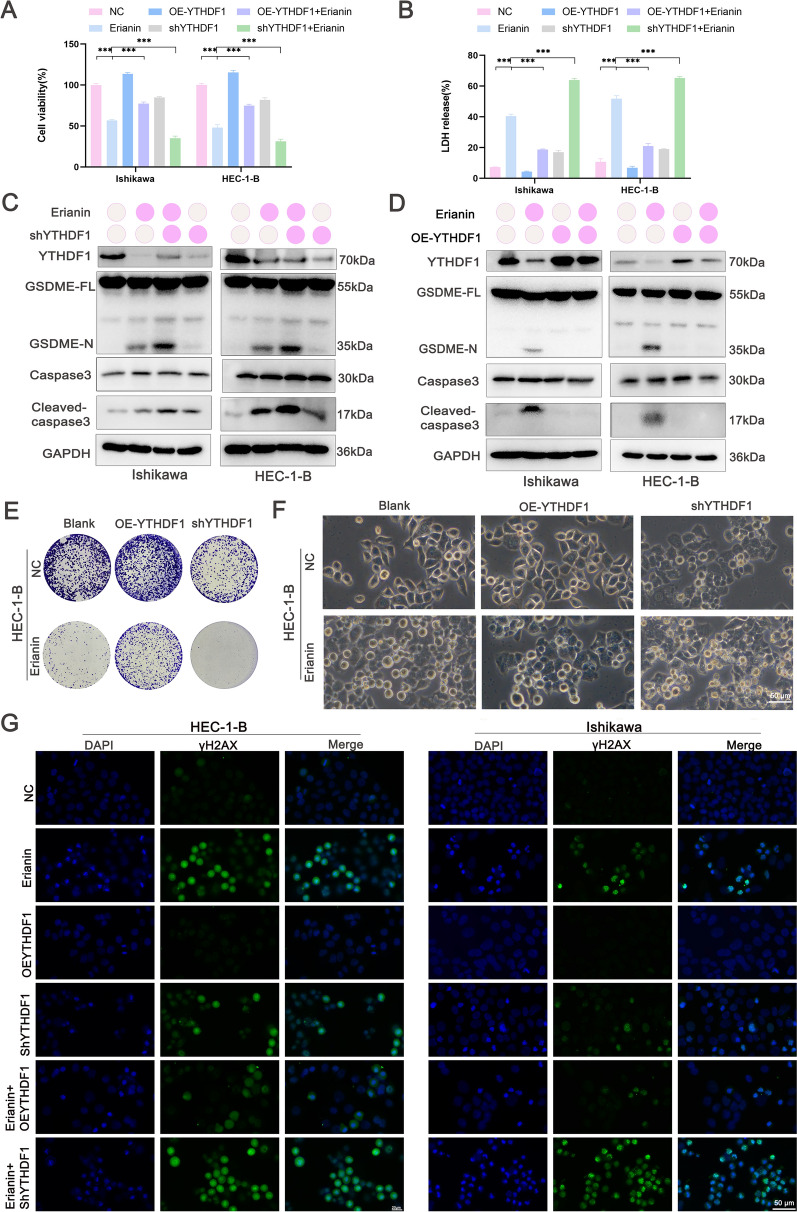
Erianin-induced EC pyroptosis depends on YTHDF1. EC cells were transfected with shYTHDF1 and YTHDF1 vectors. **A** Survival of EC cells incubated with erianin (100 nM) for 24 h, as detected using CCK-8 assays. **B** LDH release assay. **C**, **D** Western blots of YTHDF1, cleaved GSDME and cleaved caspase-3 in EC cells. **E** Colony formation assay. **F** Phase-contrast microscopy images of morphological features. Scale bar, 50 μm. **G** Representative images of γ-H2AX staining with immunofluorescence. Scale bar, 50 μm. The data are presented as the means ± SD; n = 3, ***P < 0.001 compared with the NC (control group)

The role of YTHDF1 in erianin-induced pyroptosis was investigated. YTHDF1 overexpression increased cell viability and reduced LDH release. Conversely, YTHDF1 knockdown decreased cell viability and increased LDH release (Fig. 7A, B). Western blot analysis indicated that YTHDF1 knockdown increased the levels of cleaved caspase-3 and GSDME (Fig. 7C), whereas YTHDF1 overexpression suppressed their expression (Fig. 7D). Silencing YTHDF1 impaired the colony formation ability of EC cells, whereas YTHDF1 overexpression restored it (Fig. 7E and Figure S2A). Combining YTHDF1 knockdown with erianin treatment substantially increased the proportion of swollen EC cells (Fig. 7F and Figure S2B) and DNA damage foci (Fig. 7G and Figure S2C). Conversely, YTHDF1 overexpression reduced both pyroptotic cells (Fig. 7F and Figure S2B) and DNA damage foci (Fig. 7G and Figure S2C). These findings suggest that erianin exerts anti-cancer effects by directly targeting YTHDF1.

### The stability of FOXM1 mRNA in EC cells is regulated by YTHDF1

**Fig. 8:**
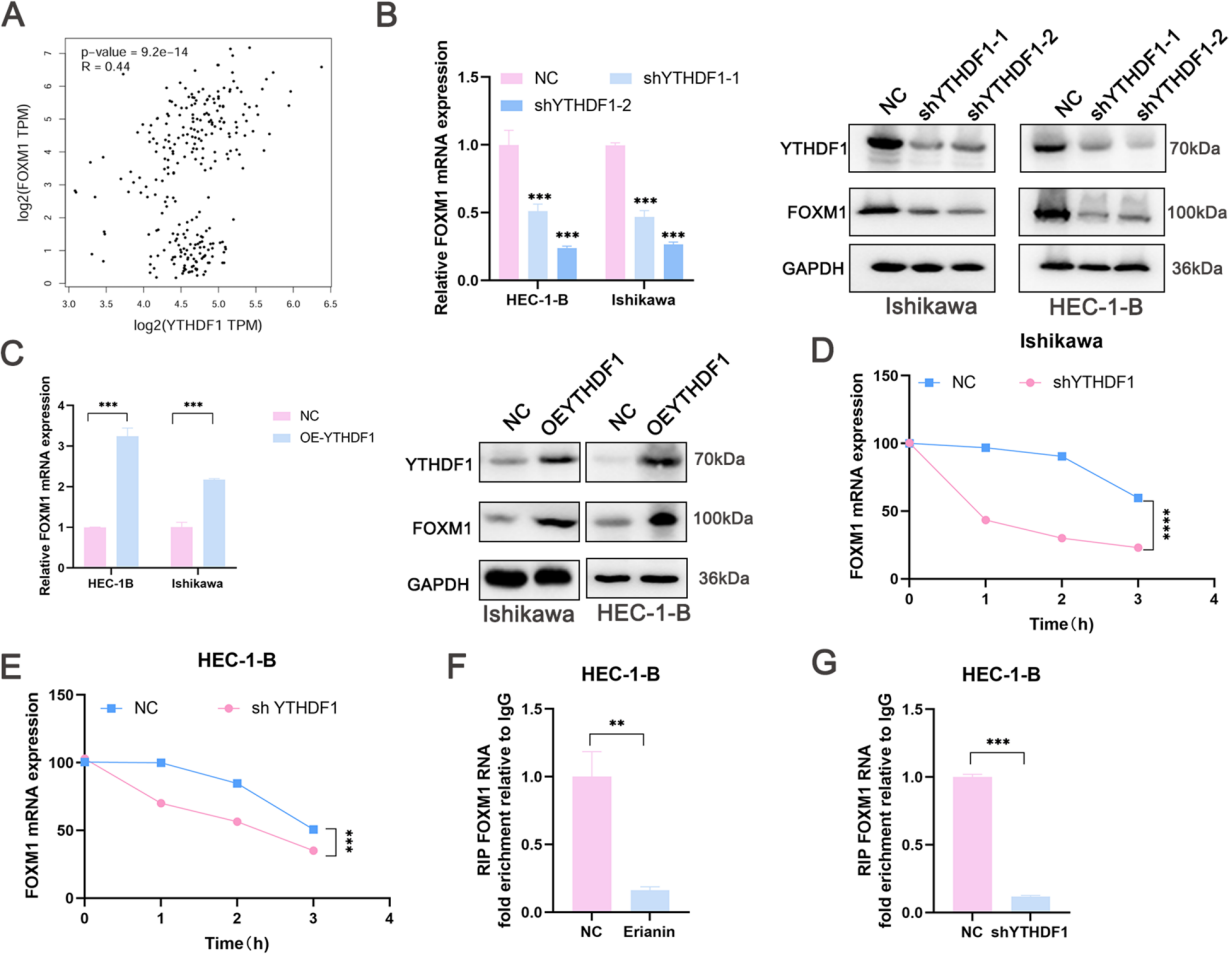
YTHDF1 regulates FOXM1 mRNA stability in EC. **A** Correlation between YTHDF1 mRNA and FOXM1 mRNA using the GEPIA database. **B** qRT‒PCR and western blotting experiments were used to validate FOXM1 expression in EC cells transfected with shYTHDF1. **C** qRT‒PCR and western blotting experiments were used to validate FOXM1 expression in EC cells transfected with shYTHDF1. **D**, **E** Relative expression of FOXM1 in Ishikawa and HEC-1-B cells with silenced YTHDF1 incubated with actinomycin D (5 μg/mL). **F** Results of the RIP assays showing YTHDF1-FOXM1 mRNA binding in the HEC-1-B cells incubated with erianin. **G** Results of the RIP assays showing YTHDF1-FOXM1 mRNA binding in the HEC-1-B cells with silenced YTHDF1. The data are presented as the mean ± SD; n = 3, *P < 0.05, **P < 0.01, and ***P < 0.001 compared with the NC (control group)

YTHDF1 recognizes m6A-modified FOXM1 and affects its stability [35]. A strong positive correlation between YTHDF1 and FOXM1 mRNA levels in the GEPIA database in EC was identified (Fig. 8A). Whether erianin regulate FOXM1 expression was determined by targeting YTHDF1. FOXM1 expression after YTHDF1 knockdown and overexpression was analyzed using qRT‒PCR and western blotting. The knockdown and overexpression of YTHDF1 inhibited (Fig. 8B) and promoted (Fig. 8C) FOXM1 RNA and protein expression, respectively. Inhibiting transcription with actinomycin D showed that YTHDF1 knockdown promoted FOXM1 degradation in EC cells (Fig. 8D and 8E). Ribonucleic acid immunoprecipitation (RIP) assays indicated that YTHDF1 knockdown (Fig. 8F) and erianin impaired YTHDF1–FOXM1 mRNA binding (Fig. 8G). These results suggest that YTHDF1 plays an important role in regulating FOXM1 expreesion.

### Effects of erianin on EC xenograft tumors in vivo

**Fig. 9:**
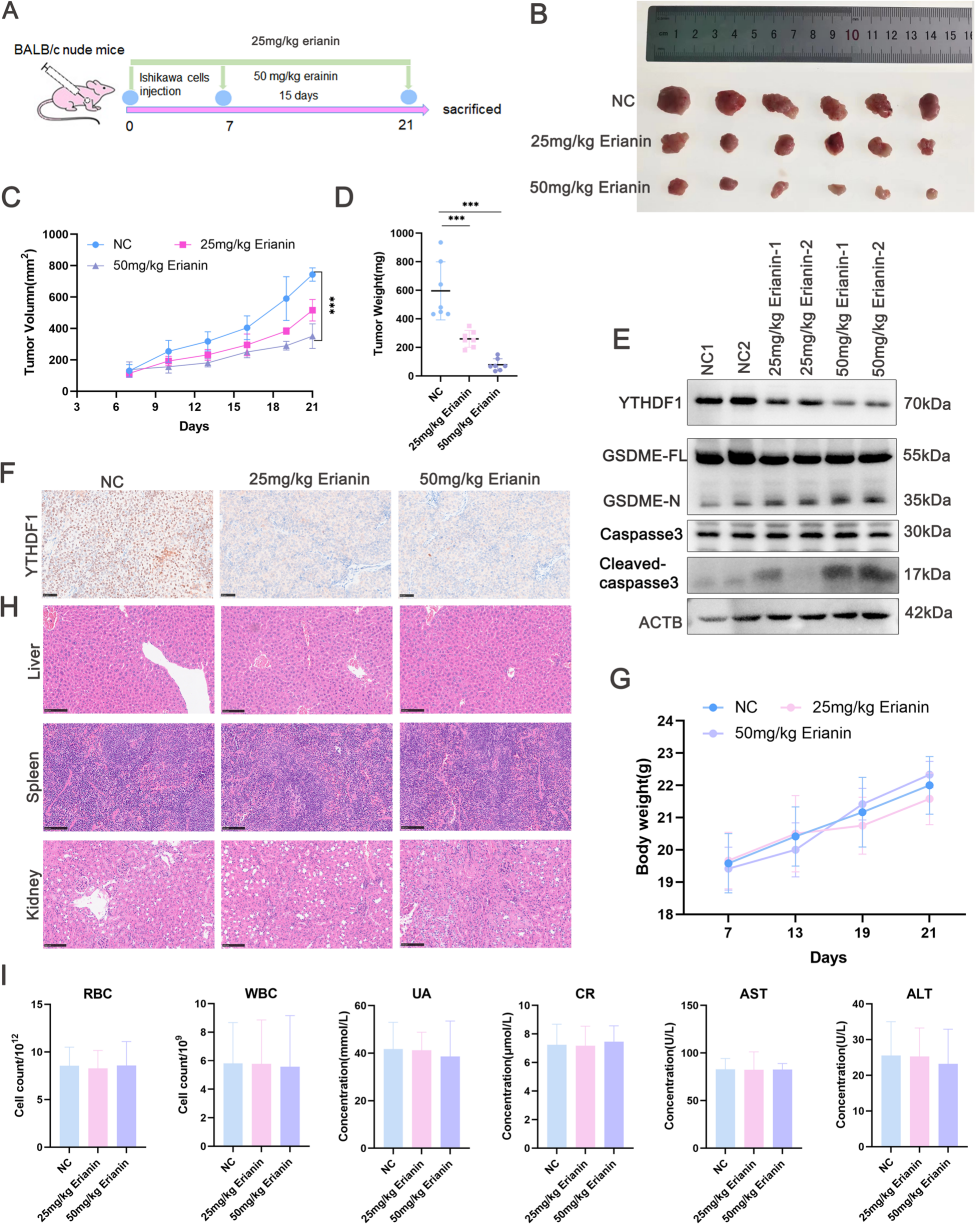
Erianin suppresses EC tumour growth in vivo. **A** Scheme of the animal experiments. **B** Erianin inhibits subcutaneous xenograft growth. (n = 6). **C** Tumor growth curves showing the effects of erianin on EC tumor xenograft growth (n = 6). **D** Tumor weight. **E** The protein levels of YTHDF1, caspase-3, cleaved-caspase3, and GSDME in tumor tissues. **F** Immunohistochemistry (IHC) staining of YTHDF1 in xenografts. Scale bar, 50 μm. **G** Weight evaluation of mice under Erianin treatment. **H** Representative images of livers, spleens, and kidneys stained with hematoxylin and eosin (H&E) at the end of the study. Scale bar, 100 μm. **I** Blood sample biochemical analysis: red blood cells (RBC); white blood cells (WBC); urea (UA); creatinine (CR); aspartate aminotransferase (AST), and alanine aminotransferase (ALT). The data are presented as the means ± SD, n = 6, ***P < 0.001 compared to the NC (control group); ns, not significant

The anticancer effects of erianin were investigated in an EC xenograft model. Ishikawa cells were inoculated into the scapulae of female BALB/c-nu nude mice. The mice were then assigned to three groups that were intraperitoneally injected with 25 or 50 mg/kg erianin or normal saline daily (Fig. 9A). Erianin markedly inhibited tumor growth and tumor weight by > 50% compared to the control (Fig. 9B‒D). The expression of YTHDF1 and caspase3/GSDME axis in tumor tissues were determined. Erianin downregulated the levels of YTHDF1 in tumor tissues (Fig. 9E, F). The upregulated levels of cleaved caspase-3 and N-GSDME were observed in erianin-treated tumors (Fig. 9E). To assess the in vivo side effects of erianin, we monitored body weight changes throughout the treatment period. No significant weight loss was observed in the mice (Fig. 9G). H&E staining of major organs revealed that no obvious pathological lesions in the liver, spleen, and kidneys of mice treated with erianin (Fig. 9H). We further analyzed the previously collected blood samples from each group for biochemical toxicity evaluation, including hematological analysis and evaluation of liver and kidney function. Parameters such as red blood cell count (RBC), white blood cell count (WBC), creatinine, urea, aspartate aminotransferase (AST), and alanine aminotransferase (ALT) in the erianin-treated groups all remained within normal ranges (Fig. 9I). These findings highlight the safety and effectiveness of erianin in treating EC.

## Discussion

Conventional platinum-based chemotherapy remains a cornerstone in the management of recurrent EC. However, its efficacy is often limited by chemoresistance and dose-limiting toxicities, emphasizing the urgent need for novel therapeutic agents [36, 37]. In recent years, natural, small-molecule compounds have attracted considerable interest in anticancer drug discovery due to their low toxicity and diverse pharmacological profiles [38]. Erianin has emerged as a promising candidate drug [39]. Erianin exerts broad-spectrum anticancer effects by targeting multiple signaling pathways. Erianin modulates metabolic reprogramming to suppress tumor growth—for instance, by disrupting pyrimidine metabolism in lung cancer [40] and impairing cholesterol metabolism in triple-negative breast cancer [41]. Erianin also promotes tumor cell death by regulation of the MAPK/ERK [42], JAK/STAT3 [43], and PI3K/AKT [44] pathways. Furthermore, erianin effectively inhibits tumor spheroid formation in pancreatic cancer models and downregulates key stemness markers, such as CD44, CD133, and SOX2, suggesting a potential strategy to overcome therapy resistance [45]. Erianin substantially suppresses the proliferation of oxaliplatin-resistant CRC cells, supporting its potential application in platinum-based chemotherapy [46]. These findings indicate that erianin could be an effective anticancer drug with substantial prospects for development. However, the role and molecular mechanisms of erianin in EC are unclear. Therefore, investigating the anti-EC effects of erianin and elucidating its potential mechanisms could promote its clinical application in EC treatment. In the current study, erianin was found to induce pyroptosis in EC cells. Some chemotherapeutic and natural compounds induce tumor cell pyroptosis via the caspase-3/GSDME axis. For example, Salvia miltiorrhiza root extracts, miltirone [47] and myricetin [48], induce cancer cell pyroptosis through the caspase-3/GSDME pathway. The current results indicate that erianin induces GSDME cleavage via caspase-3 activation, which is impeded by the silencing and inhibition of caspase-3. Using the 20 k HuProt human proteome microarray, YTHDF1, an m6A reader, was identified as the primary target of erianin. The critical role of YTHDF1 in erianin-mediated pyroptosis was investigated. RNA-seq analysis indicated that erianin regulated the FOXM1/RRM2-mediated DNA damage pathway. Inhibition of FOXM1 led to the activation of cleaved caspase-3, whereas overexpression of FOXM1 partially rescued erianin-induced pyroptosis. Mechanistically, erianin targeted and ubiquitinated YTHDF1 directly to regulate the stability of FOXM1 mRNA, thereby activating caspase-3/GSDME- dependent pyroptosis.

M6A modification plays a crucial role in tumor progression [49, 50]. The ability to target m6A has attracted considerable attention. Natural compounds can target m6A to exert anticancer effects. Cucurbitacin B (CuB) targets IGF2BP1 (Cys253), which blocks c-myelocytomatosis (c-MYC) recognition to induce apoptosis and antitumor immunity in hepatocellular carcinoma [51]. Curcumin inhibits the expression of the m6A demethylase, ALKBH5, leading to increased m6A methylation of TRAF4 and inhibition of adipogenesis [52]. YTHDF1 has also been shown to exhibit anticancer effects in various tumors. For example, the m6A reader, YTHDF1, regulates ARHGEF2 translation in an m6A-dependent manner, promoting CRC progression [53]. YTHDF1 enhances m6A-dependent TINAGL1 translation, driving malignant progression in esophageal cancer [54]. YTHDF1 mediates m6A modification of GLS1, contributing to oxaliplatin resistance in CRC [55]. YTHDF1 inhibition promotes caspase-3-dependent apoptosis [56] and enhances DNA damage [57]. Targeting YTHDF1 shows great promise in cancer therapy. Ebselen suppresses breast cancer progression by inhibiting the YTHDF1–c-Fos axis [58]. Targeting YTHDF1 enhances anti-tumor immunity and augments the efficacy of anti-PD-1 therapy in CRC [59]. Therefore, these findings establish YTHDF1 as an attractive therapeutic drug target in cancer. Erianin was identified as a potential YTHDF1-targeting compound, a finding validated by molecular docking and CETSA experiments. The current study determined that YTHDF1 was highly expressed in EC. Silencing YTHDF1 inhibited the FOXM1/RRM2 axis, enhanced DNA damage response. Erianin-induced pyroptosis in EC cells was impeded by YTHDF1 overexpression, indicating that YTHDF1 plays a pivotal role in erianin-induced pyroptosis.

The transcription factor, FOXM1, regulates various signaling pathways [60] and is upregulated in breast [61], gastric [62], pancreatic [63], and hepatocellular [64] cancers. It functions in tumorigenesis through its role in cell cycle, DNA damage, cellular senescence, and metastasis. FOXM1 regulates ZIC2, which promotes the malignant phenotype of renal clear cell carcinoma [65]. In lung cancer, FOXM1 directly binds to the promoter of RRM2, thus regulating cell proliferation and metastasis [66]. FOXM1 has also been demonstrated to play a critical oncogenic role in EC [67]. RNA-seq indicated that erianin modulates the DNA damage response and downregulates the expression of FOXM1, a key gene involved in DNA repair. The qRT‒PCR and western bloting analysis confirmed that erianin substantially downregulated FOXM1. The most differentiated target in the regulatory network of FOXM1 is RRM2, which is abundantly expressed in many types of cancers. Ribonucleotide reductase regulatory subunit M2 catalyzes deoxyribonucleotide synthesis, regulates nucleotide metabolism (deoxyribonucleotide triphosphate pools), and is closely associated with the cell cycle, chemotherapy resistance, and DNA repair mechanisms [68–70]. Unstable dNTP pools can lead to DNA damage. Oxoglutarate dehydrogenase L (OGDHL) downregulates nucleotide metabolism in hepatocellular carcinoma, which leads to dNTP depletion; this is thought to be a major factor mediating DNA damage [71]. Erianin inhibited the FOXM1/RRM2 axis to regulate DNA damage in EC cells, and knockdown of FOXM1 suppressed RRM2 expression, promoted DNA damage, and activated cleaved caspase-3 in EC cells. Overexpression of FOXM1 reverses erianin-induced pyroptosis in EC. Thus, the FOXM1/RRM2 pathway is vital to the anticancer effect of erianin in EC. Additionally, YTHDF1 binds to m6A-modified FOXM1 and promotes its expression [72]. Therefore, we hypothesize that during erianin-induced pyroptosis in EC cells, inhibition of YTHDF1 leads to decreased stability of FOXM1 mRNA. Knockdown of YTHDF1 suppresses the FOXM1/RRM2 signaling pathway, thereby inducing DNA damage and activating caspase-3/GSDME-mediated pyroptosis.

## Conclusions

A low dose of erianin robustly inhibited the progression of EC via caspase-3/GSDME-dependent pyroptosis. Erianin directly targeted YTHDF1 and promoted its ubiquitination and degradation, which induced DNA damage mediated by FOXM1/RRM2, resulting in GSDME cleavage by cleaved caspase-3. The mechanism of the anticancer effects of erianin were determined. The findings provide a theoretical basis for the development of pyroptosis-targeted anticancer drugs and new strategies with which to treat EC.

## Supplementary Information

Additional file 1

Additional file 2

## Data Availability

The datasets used and/or analysed during the current study are available from the corresponding author on reasonable request.

## Abbreviations

RBC: Red blood cells
WBC: White blood cells
DAPI: 4′,6-Diamidino-2-phenylindole
EdU: 5-Ethynyl-2'-deoxyuridine
ALKBH5: Alkylation repair homolog protein 5
AST: Aspartate aminotransferase
ALT: Alanine aminotransferase
ANOVA: Analysis of variance
AURKA: Aurora kinase A
AURKB: Aurora kinase B
BSA: Bovine serum albumin
AM: Calcein–acetoxymethyl
Z-DEVD-FMK: Cell-permeable fluoromethyl ketone
CETSAs: Cellular thermal shift assays
CENPA: Centromere protein A
CQ: Chloroquine
CRC: Colorectal cancer
JNK: C-Jun N-terminal kinases
c-MYC: C-myelocytomatosis
CuB: Cucurbitacin B
CHX: Cycloheximide
CMV: Cytomegalovirus
DNB: DNA nanoball
DEGs: Differentially expressed genes
MEK2: Dual specificity mitogen-activated protein kinase kinase 2
EC: Endometrial cancer
ECL: Enhanced chemiluminescence
FTO: Fat mass and obesity-associated protein
Cy5-SA: Fluorescence-labeled streptavidin
FOXM1: Forkhead Box M1
GSDME: Gasdermin E
GEPIA: Gene Expression Profiling Interactive Analysis
H&E: Hematoxylin and eosin
HK2: Hexokinase II
HRP: Horseradish peroxidase
IHC: Immunohistochemical
IP: Immunoprecipitation
IGF2BPs: Insulin-like growth factor 2 mRNA-binding proteins
FIGO: International Federation of Gynecology and Obstetrics
LDH: Lactate dehydrogenase
mTOR: Mammalian target of rapamycin
METTL3/14: Methyltransferase-like 3/14
METTL3: N6-adenosine-methyltransferase 70 kDa subunit
m6A: N6-methyladenosine
OGDHL: Oxoglutarate dehydrogenase L
PBS: Phosphate-buffered saline
PI3K: Phosphatidylinositol 3-kinase
PLK1: Polo-like kinase 1
PARP: Poly(ADP-ribose) polymerase
PI: Propidium iodide
qRT-PCR: Quantitative real-time polymerase chain reaction
RIPA: Radio-immunoprecipitation assay
c-Raf: RAF proto-oncogene serine/threonine-protein kinase
M2RRM2: Ribonucleoside-diphosphate reductase subunit
RRM2: Ribonucleotide reductase M2
RIP: RNA immunoprecipitation
RCA: Rolling circle amplification
shRNA: Short hairpin RNA
STR: Short tandem repeat
siRNA: Small interfering RNA
SDS‒PAGE: Sodium dodecyl sulphate–polyacrylamide gel electrophoresis
SD: Standard deviation
TOP2A: Topoisomerase IIα
TEM: Transmission electron microscopy
TBST: Tris-buffered saline with Tween-20
WTAP17: Wilms' tumor 1-associating protein
YTHDF1–3: YTH domain family proteins
