# Supplementary material for: Erianin facilitates pyroptosis in endometrial cancer via targeting m6A reader YTHDF1
Source: Chin Med. 2026 Apr 7;21:112. doi: 10.1186/s13020-025-01313-9 (PMC13054981; doi:10.1186/s13020-025-01313-9)
Supplement: Supplementary file 1 — Additional file 1 [file 13020_2025_1313_MOESM1_ESM.docx]

**FigureS1:**


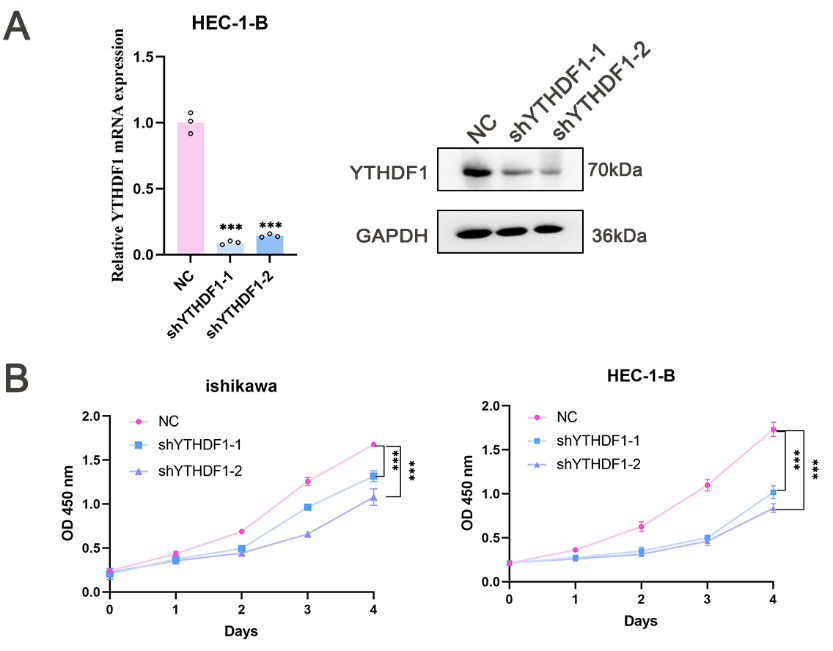


(A) RT-PCR and western blotting were used to validate the gene expression efficiency in HEC-1B cells transfected with siRNA. (B) Proliferation of EC cells transfected with shYTHDF1. Data are presented as mean ± SD; ***P<0.001 compared to NC (control group).

**FigureS2:**

**
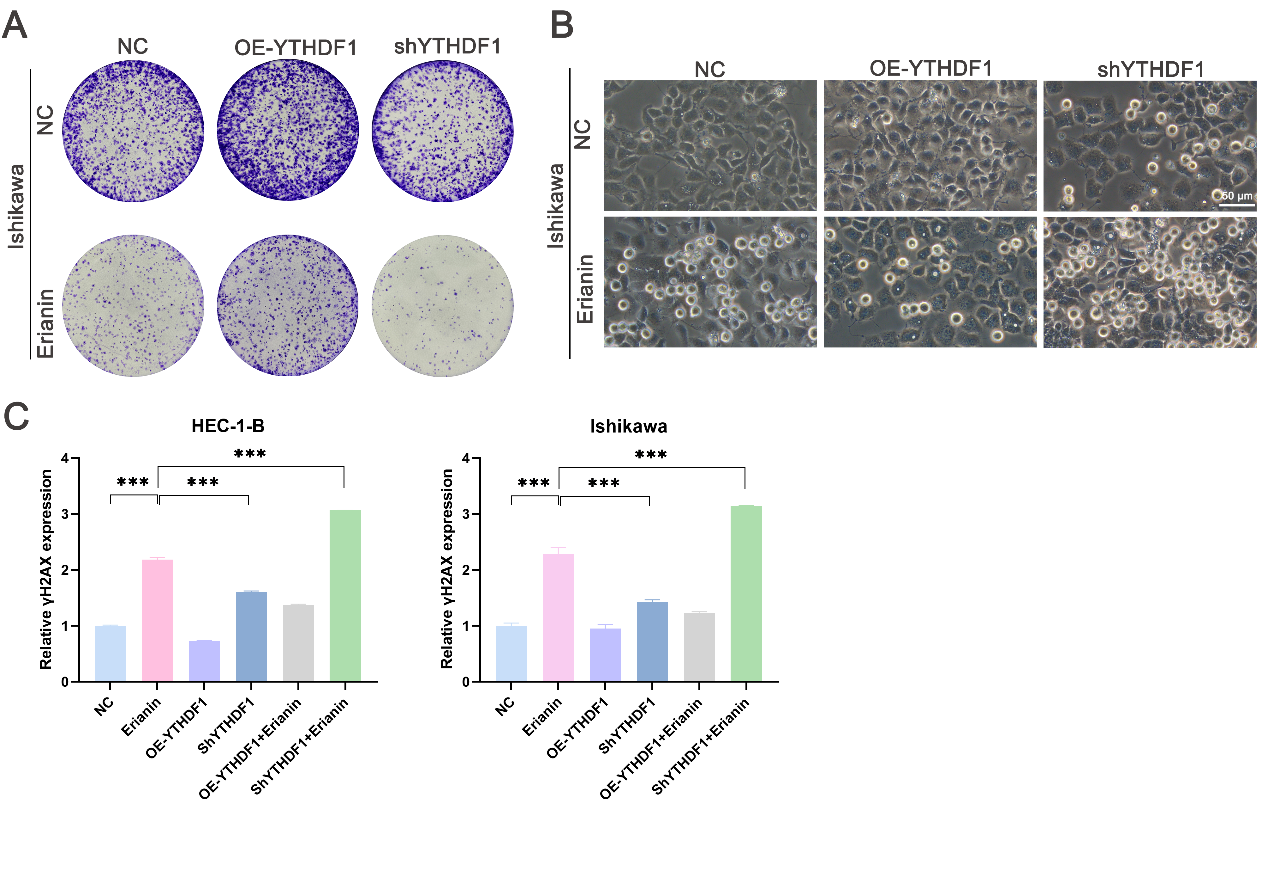
**

(A) The colony formation ability of YTHDF1 depleted and YTHDF1 overexpressed Ishikawa cells treated with erianin. (B) Observation of morphological features under a phase-contrast microscope in YTHDF1 depleted and YTHDF1 overexpressed Ishikawa cells treated with erianin. Scale bar = 50 μm. (C) Quantification of fluorescent γH2AX expression. Data are presented as means ± SD; ***P<0.001 compared to NC (control group).
